# Understanding Why Many People Experiencing Homelessness Reported Migrating to a Small Canadian City: Machine Learning Approach With Augmented Data

**DOI:** 10.2196/43511

**Published:** 2023-05-02

**Authors:** Chandreen Ravihari Liyanage, Vijay Mago, Rebecca Schiff, Ken Ranta, Aaron Park, Kristyn Lovato-Day, Elise Agnor, Ravi Gokani

**Affiliations:** 1 Department of Computer Science Faculty of Science and Environmental Studies Lakehead University Thunder Bay, ON Canada; 2 Department of Health Sciences Faculty of Health and Behavioural Sciences Lakehead University Thunder Bay, ON Canada; 3 The District of Thunder Bay Social Services Administration Board Thunder Bay, ON Canada; 4 School of Social Work Faculty of Health and Behavioural Sciences Lakehead University Thunder Bay, ON Canada

**Keywords:** data augmentation, feature selection, homelessness, machine learning, migrants

## Abstract

**Background:**

Over the past years, homelessness has become a substantial issue around the globe. The largest social services organization in Thunder Bay, Ontario, Canada, has observed that a majority of the people experiencing homelessness in the city were from outside of the city or province. Thus, to improve programming and resource allocation for people experiencing homelessness in the city, including shelter use, it was important to investigate the trends associated with homelessness and migration.

**Objective:**

This study aimed to address 3 research questions related to homelessness and migration in Thunder Bay: What factors predict whether a person who migrated to the city and is experiencing homelessness stays or leaves shelters? If an individual stays, how long are they likely to stay? What factors predict stay duration?

**Methods:**

We collected the required data from 2 sources: a survey conducted with people experiencing homelessness at 3 homeless shelters in Thunder Bay and the database of a homeless information management system. The records of 110 migrants were used for the analysis. Two feature selection techniques were used to address the first and third research questions, and 8 machine learning models were used to address the second research question. In addition, data augmentation was performed to improve the size of the data set and to resolve the class imbalance problem. The area under the receiver operating characteristic curve value and cross-validation accuracy were used to measure the models’ performances while avoiding possible model overfitting.

**Results:**

Factors predicting an individual’s stay duration included home or previous district, highest educational qualification, recent receipt of mental health support, migrating to visit family or friends, and finding employment upon arrival. For research question 2, among the classification models developed for predicting the stay duration of migrants, the random forest and gradient boosting tree models presented better results with area under the receiver operating characteristic curve values of 0.91 and 0.93, respectively. Finally, home district, band membership, status card, previous district, and recent support for drug and/or alcohol use were recognized as the factors predicting stay duration.

**Conclusions:**

Applying machine learning enables researchers to make predictions related to migrants’ homelessness and investigate how various factors become determinants of the predictions. We hope that the findings of this study will aid future policy making and resource allocation to better serve people experiencing homelessness. However, further improvements in the data set size and interpretation of the identified factors in decision-making are required.

## Introduction

### Background

Homelessness is an enduring challenge experienced by diverse populations across the world. Addressing and preventing homelessness is critical because it negatively affects the physical and mental health of these populations and causes substantial costs to the public [1]. Many important facets of homelessness have been studied by researchers, including homelessness experienced by youth, family, and veterans [2-6], as well as homelessness because of substance use [7,8], financial strain [9], mental health [9,10], and racism [11].

Homelessness in Canada has intensified rapidly over the last few decades, and many substantial changes have been identified, such as the rise in chronic homelessness and a change in the demographic representation of people experiencing homelessness from mostly single older men to families, women, and youth [12]. According to a point-in-time (PiT) count conducted in 2018 in Thunder Bay, Ontario, Canada, 77.2% of the people experiencing homelessness in the city were couch surfing or staying at emergency shelters, and the primary reason for becoming homeless was identified as addiction or substance use [13]. Moreover, the District of Thunder Bay Social Services Administration Board (TBDSSAB) has observed that the majority of the people experiencing homelessness in the city are from outside of the city or province; in fact, the 2018 PiT count found that approximately 3 in 5 people experiencing homelessness were from outside of the city, whereas approximately 1 in 5 were from outside of the province [13,14].

Because of this observed trend in migration to Thunder Bay, the TBDSSAB wanted to understand homeless mobility to optimize its resource allocation and program planning in addressing homelessness in the city. Consequently, this study presents the use of machine learning to help understand why a high proportion of people experiencing homelessness in a small Canadian city are migrants from outside of the city or province. To achieve this broader aim, we developed the following 3 research questions (RQs):

- RQ1: What factors predict whether an individual stays or leaves shelters in Thunder Bay?
- RQ2: If an individual stays, how long are they likely to stay?
- RQ3: What factors predict stay duration?

In the literature, important attention has been given to understanding homelessness. Many previous studies have presented the use of applied statistical analysis to produce qualitative results, such as finding the important risk factors contributing to homelessness [15]. Meanwhile, several studies have used machine learning concepts to perform predictive analysis and develop decision support tools related to homelessness research, such as predicting whether a person will become homeless, the duration of homeless stay [16], the readmission to the homeless state [16,17], and which individuals get housing and daily sheltering arrangements [6,18]. Furthermore, researchers have also focused on understanding homelessness by group identities, such as racial identity [11], adolescence [6,19,20], substance use [8], the uptake of medical treatments [8], and veterans [2]. However, the relationship between homelessness and migration has rarely been investigated, and no machine learning approaches have been used in this context. We used 2 machine learning models and 2 feature selection techniques to identify the patterns and make predictions in the migration-homelessness context.

The subsequent sections of this paper are organized as follows. The Literature Review subsection provides a brief review of previous studies focused on factors associated with homelessness and the application of machine learning to understanding homelessness. The *Methods* section describes our data set and the methodology behind predictive model development. The Results and Discussion sections present, respectively, the results and a discussion of the performance of various machine learning models and the findings of the study as well as a conclusion to the paper.

### Literature Review

The application of data science in addressing social science problems has become popular in recent years. As homelessness is one of the major global issues, many previous studies have conducted research to identify trends and make predictions related to homelessness [16-18]. Understanding the causal factors of homelessness has received considerable attention in the literature. The most common factors contributing to homelessness were identified as mental illness; substance use; economic status, including income; poverty; and unemployment. Other than these factors, age, sex racism, education, physical disability, family issues, domestic violence, and contact with criminal justice systems have been identified as common causal factors [8,10,11,15,21].

Although these are regarded as static factors, some studies have used dynamic factors, such as the number of stays at a shelter in a current 30-day time stamp (TS), the number of shelter meals in the current TS, and the number of shelter bed reservations in the past TSs [22]. Although these factors are valid for all regions, a study identified addiction or substance use, conflicts with the partner, and difficulty in paying house rent as the main causes of housing loss in Thunder Bay in 2018 [13]. Some studies have even analyzed the relationship between several variables and homelessness, such as the relationship among financial strain, mental illness, and homelessness [9] or the relationship among substance use, posttraumatic stress disorder, and homelessness [10].

Chronic homelessness is a substantial issue in many countries, where people enter the homeless state periodically. In 2021, a decision support system for predicting chronic homelessness among individuals in the city of London, Ontario, Canada, was developed [22]. This decision support system, which incorporated both static and dynamic attributes of a person’s history to predict chronic homelessness 6 months into the future, enabled city authorities to gain an insight into the attributes that lead to chronic homelessness. Moreover, this work used an interpretable artificial intelligence algorithm called local interpretable model-agnostic explanations to increase transparency in automated decision systems to reduce bias toward factor selection. The study developed 5 machine learning models: logistic regression, random forest (RF), Extreme Gradient Boosting (XGBoost), multilayer perceptron (MLP), and a recurrent neural network (RNN) combined with MLP called HIFIS (Homeless Individuals and Families Information System)-RNN-MLP [22]. The HIFIS-RNN-MLP model was developed to perform time-series data analysis on a dynamic data set. Three models that used a combination of static and dynamic features showed a mean recall of >90% and mean precision of >60% during the 10-fold cross-validation (CV) process. One of the main limitations of the work is that the authors used a data set from a recent 4-year period to predict chronic homelessness. Meanwhile, another study in 2021 assessed a simple threshold method with 2 predictive machine learning algorithms—logistic regression and neural network—in predicting the chronic homelessness of individuals [23]. The authors attempted to find the best model for predicting chronic homelessness and discovered that a simple threshold approach can present a performance similar to that of complex machine learning models. The main limitation of this work concerns obtaining higher positive predictions through the threshold test compared with the machine learning models. The study [24] used 2 modified deep learning algorithms—modified deep q-learning and modified neural fitted q-iteration—to find the probability of an individual moving from one homeless state to another. This work is better than a mathematical model because the probabilities are revised dynamically to produce more accurate results.

RF and logistic regression are arguably the most commonly used predictive models when applying machine learning to understanding homelessness. In predicting the reentry into homelessness, 1 study, which developed decision trees, RFs, and logistic regression models, found that the best area under the receiver operating characteristic curve (AUC) value was 0.7 [17]. The aforementioned 2 common models were used in another study to predict the persistent homelessness of specific target categories, such as recently unemployed workers and youth who entered adulthood [25]. This work was able to produce better results after experimenting with different feature selection techniques such as filter-based selection, wrapper-based forward selection and backward elimination, least absolute shrinkage and selection operator, and RF as an embedded model for feature selection. Moreover, predicting the homelessness and housing instability of veterans has been conducted using the same 2 machine learning approaches, logistic regression and RF, with the RF model producing a better performance [2]. In addition to these commonly used supervised learning approaches, the k-means algorithm has been used to identify individuals with similar stay duration and number of returns to the homeless state [16].

Another application of machine learning to understanding homelessness is in prioritizing housing assistance needs. The majority of people who receive treatment for substance use and mental illness will eventually need housing assistance. In 2020, researchers published a study that analyzed clinical records related to substance use treatment admissions and discharges to prioritize the housing requirements of patients according to the risk of becoming homeless [8]. The authors used logistic regression to predict whether a patient would be homeless by the end of treatment. In addition, they showed how certain attributes such as level of access to health care, types of mental illness, details of secondary substance use, and patient gross income are potentially important in identifying the possibility of becoming and remaining homeless. By contrast, a study has supported policy making for solving homelessness by conducting experiments on reducing the time spent on housing assistance and diminishing the inflow into homelessness assistance using simulation models [1]. The authors suggested that the reduction in inflow to the housing assistance service could lower homelessness and housing insecurity substantially. One study has developed a platform to facilitate housing opportunities for people experiencing homelessness by considering the diversity of individuals experiencing homelessness and the nature of services [26]. The authors used 8 novel heuristic algorithms to search for suitable facilities required for people experiencing homelessness. In addition, the application provides many services, such as providing an analytical tool for policy makers, homelessness growth prediction, managing homeless information, and changing the housing providers to improve the satisfaction of the homeless individuals. Other than housing assistance, models to automatically recommend IDs of homelessness service providers to individuals when they experience homelessness for the first time have been developed by some studies. Among the 3 models developed, k-nearest neighbor (KNN), RF, and multiclass AdaBoost (MA), the MA model showed better results in performing multiclass classification [27].

The key input to a homelessness-related research study is the data set. To overcome the limitation of data scarcity, many studies have used private data sets for conducting their research; for instance, some studies have conducted surveys through interviews with individuals who are currently experiencing homelessness, at risk of being homeless, or were previously homeless [11,28]. Although many studies have used private databases connected to sophisticated homelessness data management systems [15,17,22], a few previous works attempted to integrate several data sources [2,25,27]. To increase the size of the data set, 1 study simulated each member as an individual and also added a synthetic data set [24]. The impact was evident with more accurate results.

Although numerous studies have considered diverse factors causing homelessness, very few studies have investigated the relationship between migration and homelessness [29]. Moreover, to the best of our knowledge, no study has yet applied machine learning to investigate the relationship between, and trends behind, migration and homelessness. Thus, the information we provide here will be useful for our understanding of migration and homelessness because we focused on developing several machine learning models on 29 factors that are associated with migration and homelessness. In the next section, we describe our data set and the in-depth model development strategies.

## Methods

**Figure 1:**
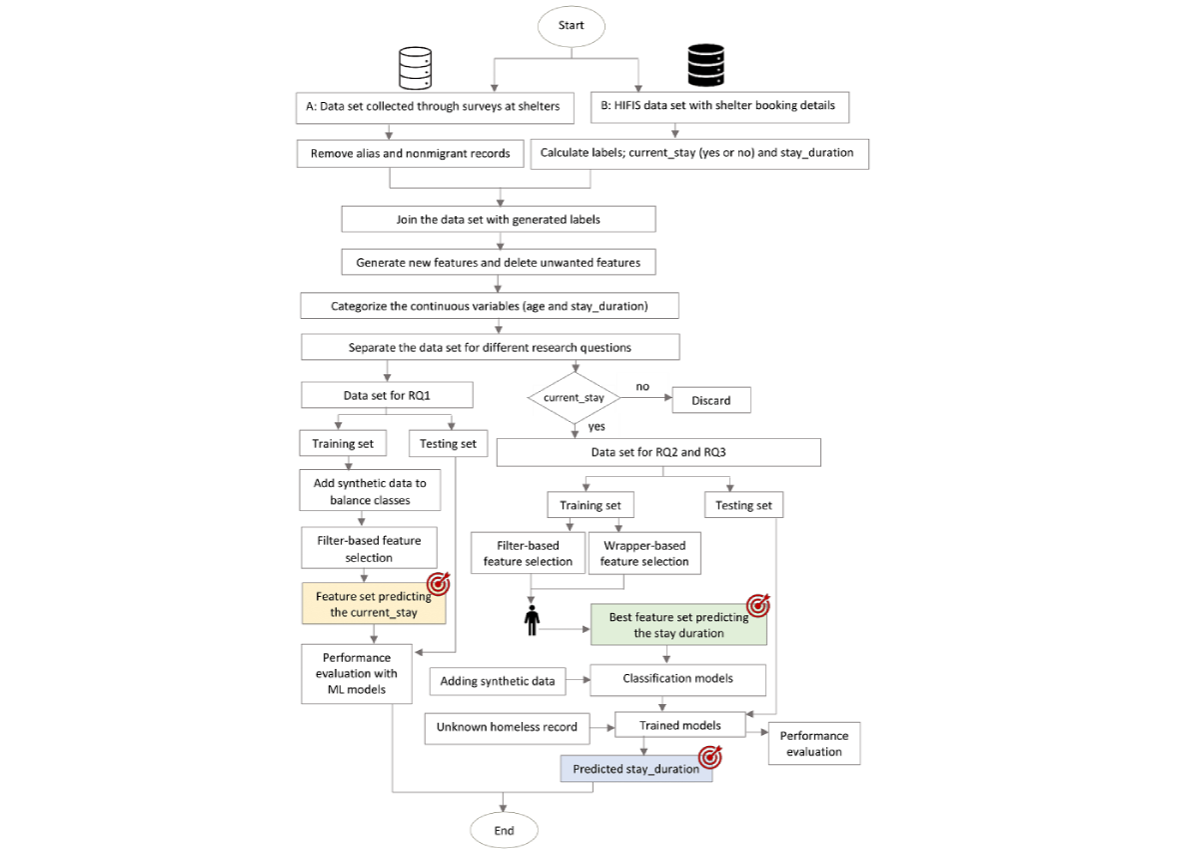
Steps in model development, including data preprocessing, feature selection, model development, and achieving objectives. HIFIS: Homeless Individuals and Families Information System; ML: machine learning; RQ: research question.

In this section, we describe the tools and methods used for the data collection and model building. Figure 1 shows the important stages of the study.

### Data Collection

The data set was gathered from 2 sources. The first source was a survey administered to people experiencing homelessness at 3 shelters across Thunder Bay: Shelter House, Salvation Army, and Urban Abbey. Adults experiencing homelessness registered at any of these shelters were surveyed weekly on Fridays from October 29, 2021, to April 9, 2022. The survey was administered using a web-based format (Multimedia Appendix 1) and consisted of 33 questions related to risk factors associated with homelessness. Only people experiencing homelessness who met the sole selection criterion—that they were from outside of the city or province—were asked to fill out the survey. The second source of data was the HIFIS, which is a federally sourced case management system designed to help better understand homelessness at the regional level. In Thunder Bay, the HIFIS is managed by the TBDSSAB, which provided shelter stay–related data such as booked-in dates, booked-out dates, and registered dates at shelters. After collecting the survey data and the variables from the HIFIS, the 2 sources of data were integrated into a single data set.

**Figure 2:**
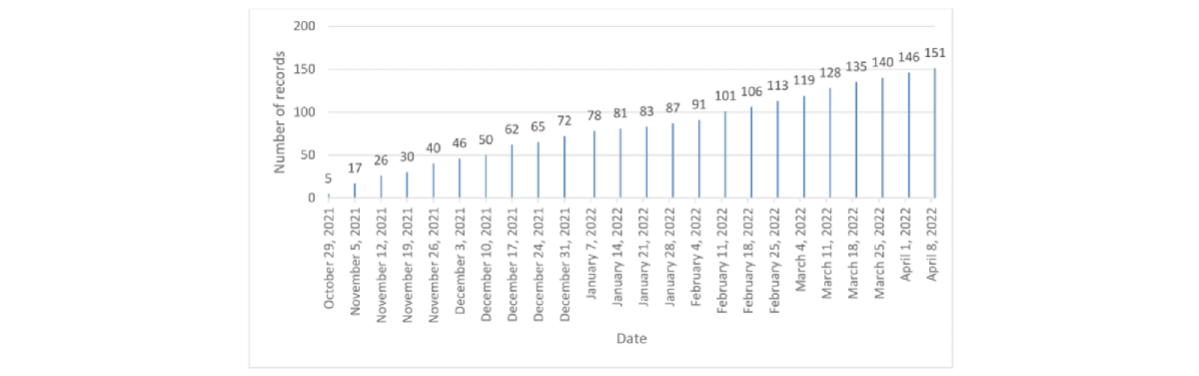
Total number of data records throughout the survey period.

The total number of records obtained at the end of the survey period was 151. The weekly distribution of the total number of unique individuals is presented in Figure 2; on average, we received 6 (SD 2.7) data points per week.

### Data Preprocessing

The data set was preprocessed before we used the data in the machine learning models. First, of the 179 records, we removed the alias records (n=28, 15.6%) because some of the people had provided their names incorrectly; next, we removed records of some of the individuals whose previous community was Thunder Bay because they were considered nonmigrants (41/151, 27.2%). Thus, of the 151 records, 110 (72.8%) remained for analysis, representing 110 unique individuals who had migrated to Thunder Bay. Meanwhile, using the HIFIS, we generated two new columns: (1) current stay: a binary column representing whether the person had stayed in shelters at the time of data collection and (2) stay duration: the number of days between their booked-in and booked-out dates. We used these 2 columns as the dependent variables. For the *current stay* calculation, the label was set to *no* if the total duration a person was not registered at any of the shelters was ≥8 weeks; otherwise, the label was set to *yes*. This threshold duration value was finalized by experts with domain knowledge. Finally, these newly generated 2 target columns were integrated into the survey data set.

The next stage of the preprocessing concerned removing several unwanted columns from the data set. Because of many null values, 2 columns—“At what age did you come for schooling?” “What type of schooling brought you here?”—were removed. Furthermore, another column—“Reasons for not going back to the previous community”—was ignored because it contained short-form qualitative (text) data. Finally, we removed the first and last names of individuals because they were no longer useful in the work. Subsequently, 4 new columns were generated using the available factors *home community* and *previous community*. The new column labels are *home district* (the district in which the home community is located), *previous district* (the district in which the previous community is located), *community status* (representing the similarities between the migrants’ home and previous communities), and *district status* (representing the similarities between the migrants’ home and previous districts). Finally, *home community*, *previous community*, and *ethnicity* were removed from the data set because they contained a large number of categories.

**Figure 3:**
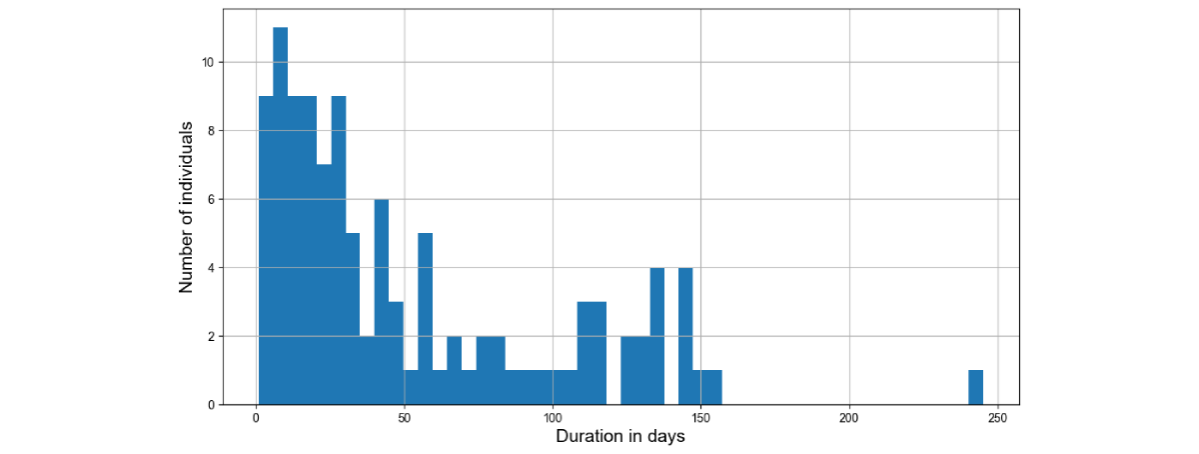
Duration of stays at shelters of individuals experiencing homelessness.

Because of the challenge involved in predicting continuous values from a small data set, the stay duration column with continuous numerical values was converted into 2 categories. The number of categories was finalized based on optimizing the model classification result. To preserve the class balance, this categorization was carried out based on the frequency binning technique to place an equal number of records into each category. The same approach was performed on the age variable to convert it into 2 categories. The original distribution of the stay duration of clients at shelters is shown in Figure 3. According to the distribution, the majority of the people experiencing homelessness have resided in shelters for a small number of days.

### Machine Learning Model Development and Hyperparameter Tunning

With regard to the 3 RQs, RQ1 concerns the factors predicting an individual’s stay or leave behavior at shelters. These factors were determined based on the binary class label *current stay.* RQ2 and RQ3 concern predicting the stay duration of an individual at shelters and identifying the factors affecting the prediction of the stay duration, respectively. These 2 questions were addressed based on the *stay duration* class label of the data set, and this too was a binary classification problem. Regarding the analysis, the total data set was used for RQ1, whereas only the records of individuals who were staying at shelters were used for RQ2 and RQ3. Of the total 110 records, the records of individuals who were staying at shelters amounted to 88 (80%).

Although RQ1 could be addressed without training classification models, we used 2 ensemble learning–based machine learning models—RF and gradient boosting tree (GBT)—to evaluate the performance of RQ1 results. For hyperparameters, the number of trees in RF and the number of boosting stages in GBT were set to a maximum of 500. For RQ2, to perform classification, we developed 8 machine learning models and conducted hyperparameter tuning to improve their performance. The types of models and their corresponding hyperparameters are listed in Textbox 1 (refer to Multimedia Appendix 2 [22,30-37] for details). Before feeding the models, both data sets (the total data set and the records of individuals who were staying at shelters) were split into training and testing sets according to 70% and 30% proportions, respectively.

For the performance evaluation, the AUC was used. The AUC is a performance measurement used in classification problems to represent the degree of separability based on the true-positive and false-positive rates of classification. The AUC values vary from 0 to 1, where the data set is perfectly separable when the value is 1 and purely inseparable when the value is 0.5 [38]. Moreover, we performed 5-fold CV on the training data set for improving and measuring the performance in terms of accuracy. Apart from this, classification reports and confusion matrices were used to evaluate the correctness of the actual and predicted labels.

**Summary of the machine learning models and their hyperparameter settings.**

- Decision tree classifier: max_depth=5 and criterion=‘gini’
- Support vector machine: kernel=‘linear,’ c=4, and probability=True
- K-nearest neighbor: n_neighbors=6
- Naïve Bayes: priors=None
- Random forest: n_estimators=500, bootstrap=True, and random_state=0
- Logistic regression: solver=‘newton-cg’ and random_state=0
- Multilayer perceptron: solver=‘adam,’ activation=‘relu,’ learning_rate=‘adaptive,’ alpha=1e-6, max_iter=500, hidden_layer_sizes=(100,), and random_state=0
- Gradient boosting tree: n_estimators=500 and random_state=0

### Feature Selection

After removing several features during the preprocessing stage, 29 independent features remained in the data set. However, because of the limited sample size, the removal of more features was required to avoid overfitting and producing inaccurate results. Furthermore, identifying the most important subset of features in predicting the *stays* or *leaves* (RQ1) and the stay duration (RQ3) of individuals at shelters was required because these are 2 of our 3 RQs. Hence, more attention was given to the feature selection techniques by following 2 main approaches: *filter-based* feature selection and *wrapper-based* feature selection [39,40].

The filter-based approach ranked the attributes based on their correlation with the target attribute. Here, the features were ranked according to the chi-square test values measured between the target and the features, and the score was high for highly correlated features. This method does not use any machine learning model for selecting the features, whereas the wrapper-based technique uses a predictive model to identify the most important features in predicting the classes.

For RQ1, we chose the filter-based feature selection technique to find the best factors predicting *stays* or *leaves* and finalized the best feature subset based on the scores and *P* values returned from the chi-square test. The results were further verified by the domain experts. For RQ3, both filter-based and wrapper-based feature selection techniques were used to find the best features predicting stay duration. The main reason for using a wrapper-based technique for improving feature selection is that RQ3 is associated with RQ2—predicting stay duration—which requires machine learning model training tasks. Moreover, because the original data points used for RQ3 are comparatively fewer (88 records) than those used for RQ1 (110 records), more attention was paid to improve feature selection. In addition, the results in RQ3 were further evaluated by a domain expert to verify the results. 

### Data Augmentation

As the data set used for RQ1 was highly imbalanced—the ratio of 2 labels was 4:1—a variant of the synthetic minority oversampling technique was first used to resample the data set, considering that these data only consisted of categorical features. Through this method, the data points of the minority class were increased to match the number of majority class points, and we were able to both achieve class balance and improve the size of the training data set to 124 for RQ1 before feature selection.

After selecting the best feature set, for both data sets, a further experiment was conducted to improve the size of the data set through data augmentation. To perform data augmentation, we used the *DataSynthesizer* approach, which considers the correlation of attributes for generating synthetic data [41]. DataDescriber and DataGenerator are 2 of the 3 high-level modules that make up the *DataSynthesizer* tool. DataDescriber analyzes the data types, correlations, and distributions of the original data set and produces a data summary, adding noise to the summarized description to preserve privacy. DataGenerator samples from the summary computed by DataDescriber and produces synthetic data samples. Moreover, *DataSynthesizer* operates in 2 modes: independent data generation and correlated data generation. We used the correlated attribute mode to generate synthetic data by considering the correlation among attributes using Bayesian networks developed based on the greedy Bayes algorithm. For developing the Bayesian network, we set several parameters, with the maximum number of parents in the network set to 3 and the epsilon set to 0 to turn off the noise injections.

### Ethics Approval, Informed Consent, and Participation

The study was approved by Lakehead University’s research ethics board (1468867; research with human participants under the government of Canada’s Tri-Council Policy Statement: Ethical Conduct for Research Involving Humans [TCPS 2; 2018]). All university researchers, including faculty and students, completed the TCPS 2 certification, indicating completion of the tutorial on research ethics. Data were collected from participants with proper consent procedures and information regarding the study, its purpose, the risks and benefits, and the participants’ rights and responsibilities, including the right to withdraw at any time up until submission of the data and the voluntary nature of participation. Participants were notified that the data would be kept confidential. There was no monetary compensation for participation.

## Results

In this section, we present the results of the experiments conducted to address each RQ.

### Factors Predicting Whether People Experiencing Homelessness Stay or Leave Shelters in Thunder Bay (RQ1)

In addressing RQ1, we needed to find the factors important for predicting whether a person experiencing homelessness was going to stay or leave the shelter. As explained in the *Methods* section, the correlation between the target variable *current stay* and the other independent features was measured through the filter-based feature selection method. Table 1 shows the most important features predicting the current stay.

As described in the *Methods* section, the 16 factors listed in Table 1 were further used to train 2 machine learning models to measure their impact on predicting whether people experiencing homelessness stay or leave shelters. As a result, the RF model obtained an AUC of 0.74 and a CV accuracy of 0.89, and the GBT model obtained an AUC of 0.73 and a CV accuracy of 0.83.

**Table 1:** Answer to research question 1: features predicting whether an individual experiencing homelessness stays or leaves the shelter.

Features	Score	*P* value
Home district	48.01	<.001
Previous district	26.84	<.001
What is the highest level of education you have completed?	22.26	<.001
Have you received any mental health support recently?	20.57	<.001
Did you find employment when you arrived in Thunder Bay?	19.70	<.001
Was your family or were your friends a reason you came to Thunder Bay?	17.86	<.001
Was education or school a reason you came to Thunder Bay?	16.20	<.001
Have you received any support for drug and/or alcohol use recently?	14.29	<.001
Was mental health support a reason you came to Thunder Bay?	13.50	<.001
Was a medical appointment for yourself or a family member a reason you came to Thunder Bay?	13.00	<.001
What is your age?	12.60	<.001
Was employment a reason you came to Thunder Bay?	9.85	.002
Was housing a reason you came to Thunder Bay?	9.00	.003
Was support for drug and/or alcohol use a reason you came to Thunder Bay?	9.00	.003
Do you have a status card?	6.00	.01
Have you been hospitalized in Thunder Bay?	5.73	.02

### Predicting the Stay Duration of Migrants at Shelters in Thunder Bay (RQ2)

Table 2 shows the best results of 8 classification models when tested against the number of features (feature experiment) and amounts of synthetic data (synthetic data experiment) using 2 different methods. The difference between these 2 methods is that the first used a filter-based feature selection approach (method 1), and the second used a wrapper-based feature selection approach (method 2). Here, CV accuracy is used to decide the best number of features and AUC values to select the best synthetic data percentage; for example, in method 1, the decision tree recorded the best feature count as 5 at a maximum CV accuracy of 0.69 and an AUC of 0.67 at a similar feature count. These 2 values can be considered intermediate performances of the model because they were further improved by adding synthetic data. Next, the model gained 186% as the best synthetic data percentage at a maximum AUC of 0.80 and a CV accuracy of 0.79 at the same synthetic percentage.

In method 1, both CV accuracy and AUC values of all models, except MLP, increased after synthetic data were added. Compared with method 1, the majority of the models in method 2 gained better CV accuracies during feature selection. In addition, many of the models selected a nearly similar number of features using the wrapper-based approach. Furthermore, in all models in method 2, except naïve Bayes, the AUC values were considerably higher after performing data augmentation. In fact, many of the models required a large amount of synthetic data to be added to achieve their best performance in both methods.

The confusion matrices in Table 3 present how the predicted and actual labels of different classification models changed in method 1 after synthetic data were added to the final data set. As demonstrated by the final results presented in Tables 2 and 3, the decision tree, RF, and GBT models outperformed the other models in predicting stay duration. Therefore, we can conclude that all tree-based models, especially the models developed based on ensemble learning, performed significantly better than the other models.

**Table 2:** Results of classification models in predicting stay duration.

Model	Method 1									Method 2						
	Feature experiment				Synthetic data experiment				Feature experiment					Synthetic data experiment		
	Best feature count from filter-based method	Intermediate performance (CV^a^ accuracy)	Intermediate performance (AUC^b^)	Best synthetic percentage		Final model performance (CV accuracy)	Final model performance (AUC)	Best feature count from wrapper-based method			Intermediate performance (CV accuracy)	Intermediate performance (AUC)	Best synthetic percentage		Final model performance (CV accuracy)	Final model performance (AUC)
DT^c^	5	0.69	0.67	186		0.79	0.80	6			0.69	0.68	162		0.75	0.90
SVM^d^	14	0.69	0.73	190		0.73	0.77	10			0.85	0.66	162		0.83	0.81
KNN^e^	13	0.62	0.69	132		0.73	0.76	19			0.70	0.61	170		0.78	0.82
NB^f^	9	0.77	0.63	191		0.78	0.66	11			0.74	0.68	75		0.81	0.69
RF^g^	25	0.74	0.60	196		0.83	0.94	14			0.72	0.65	134		0.83	0.91
LR^h^	8	0.72	0.64	65		0.76	0.66	14			0.80	0.68	131		0.78	0.82
MLP^i^	13	0.69	0.64	40		0.67	0.62	10			0.74	0.63	185		0.83	0.84
GBT^j^	18	0.66	0.58	190		0.78	0.91	11			0.67	0.60	119		0.84	0.93

^a^CV: cross-validation.

^b^AUC: area under the receiver operating characteristic curve.

^c^DT: decision tree.

^d^SVM: support vector machine.

^e^KNN: k-nearest neighbor.

^f^NB: naïve Bayes.

^g^RF: random forest.

^h^LR: logistic regression.

^i^MLP: multilayer perceptron.

^j^GBT: gradient boosting tree.

**Table 3:** Test accuracies of each model for synthetic data performance evaluation in method 1.

Model	Before data augmentation	After data augmentation
DT^a^	0.67	0.74
SVM^b^	0.63	0.70
KNN^c^	0.63	0.74
NB^d^	0.56	0.59
RF^e^	0.52	0.78
LR^f^	0.52	0.63
MLP^g^	0.48	0.56
GBT^h^	0.63	0.81

^a^DT: decision tree.

^b^SVM: support vector machine.

^c^KNN: k-nearest neighbor.

^d^NB: naïve Bayes.

^e^RF: random forest.

^f^LR: logistic regression.

^g^MLP: multilayer perceptron.

^h^GBT: gradient boosting tree.

### Factors Predicting Homeless Migrants’ Duration of Stay at Shelters in Thunder Bay (RQ3)

Similar to the outcomes of RQ1 presented in Table 1, in Table 4, we present the best predictors of the duration of an individual’s stay at a shelter in Thunder Bay using the filter-based feature selection method. Only the top 5 factors in the table are significant at *P*<.05.

Readers unfamiliar with the history of Canada and Indigenous peoples will note 2 features in the list (Tables 1 and 4) that warrant comment and that have their origins in Canada’s Indian Act, a piece of legislation pertaining specifically to Canada’s Indigenous peoples that structures the relationship between the federal government and Indigenous peoples. The Indian Act created *reserves*, portions of land designated for Indigenous peoples. Accordingly, *band membership* refers to the membership that recognizes an Indigenous person’s claim to living on a particular reserve, whereas *status card* refers to the possession of an ID card that indicates *Indian status* under the Indian Act. We should mention that these issues—the Indian Act, reservations for Indigenous peoples, and having an ID card to designate membership in an ethnic group—are all very controversial and contentious. Readers interested in knowing more about these issues can do further reading, but for the purpose of this paper, we provide this brief explanation so that readers understand what these 2 features mean.

Next, the attempt to find the important features was repeated using the wrapper-based feature selection approach. Table 5 shows the common set of best features derived from the results of different wrapper-based models, where the top rank was given to the feature that resulted from the highest number of wrapper models.

**Table 4:** Answer to research question 3: features predicting the stay duration of an individual at a shelter using the filter-based feature selection method.

Feature	Score	*P* value
Home district	6.13	.01
Do you have band membership?	5.53	.02
Do you have a status card?	4.84	.03
Previous district	4.53	.03
Have you received any support for drug and/or alcohol use recently?	4.17	.04
Did you find employment when you arrived in Thunder Bay?	1.95	.16
Have you received any mental health support recently?	1.94	.16
What is your sex?	1.79	.18
Was a medical appointment for yourself or a family member a reason you came to Thunder Bay?	1.39	.24
Community status	1.33	.25
What is your age?	1.03	.31
District status	1.00	.32
Do you feel you cannot go back to your community for any other reasons?	0.85	.36
Was support for drug and/or alcohol use a reason you came to Thunder Bay?	0.71	.40
What is the highest level of education you have completed?	0.57	.45
Have you been hospitalized in Thunder Bay?	0.49	.48

**Table 5:** Results of the wrapper-based feature selection method in predicting stay duration.

Feature	Rank based on the frequency
Did you find employment when you arrived in Thunder Bay?	1
Do you have band membership?	1
Was employment a reason you came to Thunder Bay?	2
Home district	2
Have you received any support for drug and/or alcohol use recently?	2
Was your family or were your friends a reason you came to Thunder Bay?	3
Do you have a status card?	3
Have you received any mental health support recently?	4
What is your age?	4
What is the highest level of education you have completed?	4
Previous district	5

## Discussion

In this section, the aforementioned results will be further analyzed to find answers to our 3 RQs. Moreover, the outcomes of the tests performed to find the best feature sets and synthetic percentages will be discussed in this section.

### Features Predicting Staying in or Leaving Shelters

Although all the factors listed in Table 1 met the threshold for statistical significance at *P*<.05 and resulted in better machine learning performances, we decided, with the help of domain experts, to finalize the results of RQ1 using the factors that are the strongest predictors of stay or leave at *P*<.001. The directions of the important factors were found using the Cramér’s V technique.

According to the findings, the following factors influenced whether migrants were more likely to stay at shelters:

- Received mental health support recently
- Found employment when they arrived in Thunder Bay
- Migrated to Thunder Bay for family or friends
- Migrated to Thunder Bay for education
- Received support for drug and/or alcohol use recently
- Migrated to Thunder Bay for mental health support
- Migrated to Thunder Bay for a medical appointment

In addition to these factors, those people experiencing homelessness who were older, those whose home or previous district was a neighboring one (bordering Thunder Bay), and those with some high school as the highest level of education tended to stay at shelters.

### Features Predicting the Duration of Stay at Shelters

With the help of domain experts, by considering the factors that met the threshold for statistical significance in Table 4 and the set of common features that appear in both Tables 4 and 5, we identified the following most important factors in predicting the stay duration of migrants experiencing homelessness in Thunder Bay:

- Home district
- Band membership
- Status card
- Previous district
- Support for drug and/or alcohol use

The home and previous districts of individuals experiencing homelessness become important features where the home and previous districts are the same, and it borders Thunder Bay and emerges as significant in predicting the stay duration. Moreover, individuals without a band membership and status card are also more likely to stay at shelters for longer durations. Similarly, individuals who have not received any support for drug and alcohol use recently will stay for longer periods.

### Comparison Between Filter-Based and Wrapper-Based Feature Selections

**Figure 4:**
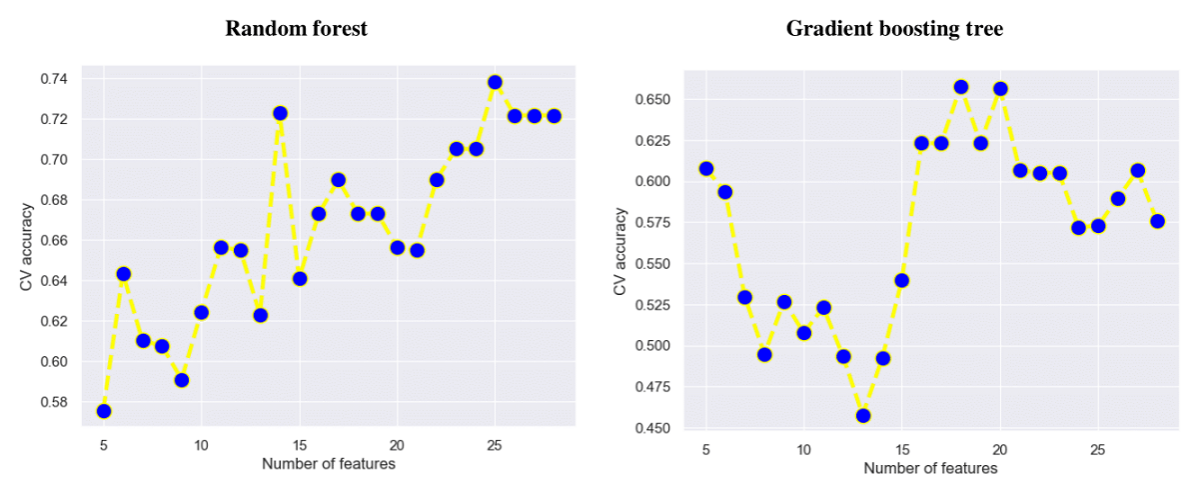
Performance plots of the 2 best classification models against different numbers of features obtained using the filter-based feature selection technique. CV: cross-validation.

Although filter-based feature selection is recognized as a faster technique than wrapper-based feature selection, its main disadvantage is that it does not consider the effect of the selected subset on the performance of the classification model when predicting the output [40]; in addition, the decision regarding best feature count is taken arbitrarily. To overcome this disadvantage, we did not use a random number of features from the ranked feature set returned by the filter-based technique as is. Instead, starting from the top-ranked feature, we added features one by one and analyzed the performance of each classification model against a different number of features. Finally, the best feature subset was chosen from the instance where the predictive model showed the best performance. Figure 4 shows how the performance of the 2 best classification models—RF and GBT—developed for predicting the stay duration changed against the different number of features obtained using the filter-based feature selection approach. As demonstrated, the performance of RF improved (CV accuracy) as the number of features increased, specifically optimized at 25 features, whereas GBT showed the best result with 18 features, with a decline in performance when the number of features was lower or higher.

In the wrapper-based feature selection approach, however, we used the feature subset returned from the wrapper-based machine learning model as the input to the classification model. In many situations, the algorithms used for classification and feature selection are the same. However, as naïve Bayes, KNN, and MLP are not supported as wrapper-based models in feature selection, the RF model was used as their wrapper instead.

### Synthetic Data in Performance Improvement

**Figure 5:**
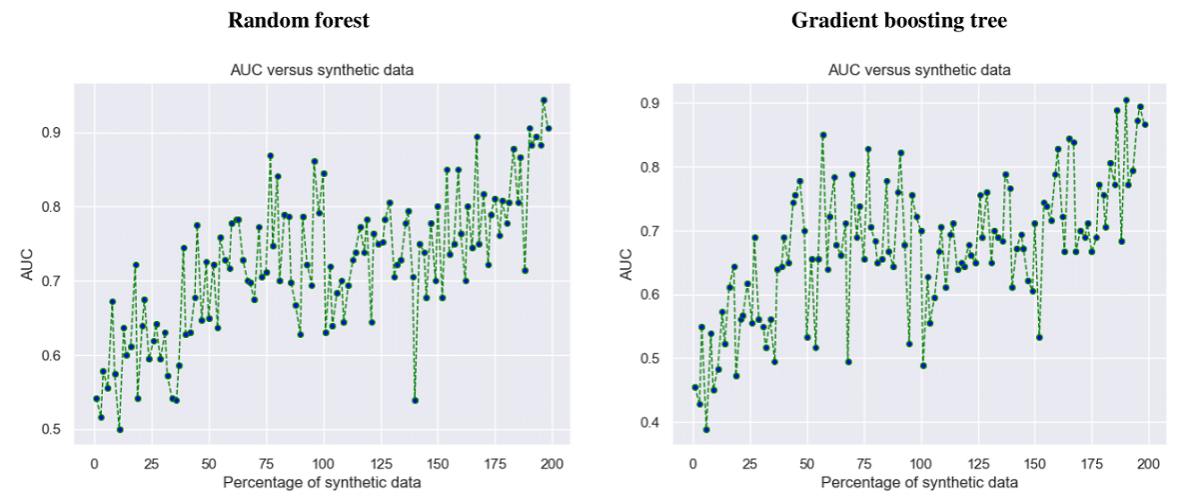
Performance of the 2 best machine learning models against different synthetic data percentages. AUC: area under the receiver operating characteristic curve.

The experiment with synthetic data was conducted by adding different percentages of synthetic data and monitoring the performance of the machine learning models. Here, up to 200% of synthetic data were added to the original data set with the best number of features. Finally, the best performance of each model was recorded along with its best synthetic data percentage. Figure 5 shows how the performances in terms of AUC values of the 2 best models (RF and GBT) changed when the proportion of synthetic data was increased in method 1.

Both plots show overall upward curves toward the incremental addition of synthetic data. Therefore, the overall results proved that the addition of synthetic data has performed an important role in improving the results of both machine learning models.

### Use of CV and Performance Evaluation

It has been proved in the literature that the k-fold CV approach produces better results for smaller data sets than other approaches, such as nested k-fold CV and the traditional training and testing split technique [30]. Moreover, the use of a complete data set for the k-fold CV can lead to overfitting and overoptimistic results [30]. Hence, we combined both the traditional training and testing split approach and k-fold CV, where only the training set was used to validate the performance through CV, and kept the testing set separate for use as a fresh data set to evaluate the models.

### Limitations and Future Improvements

The main limitation of this study is data scarcity. We attempted to offset this limitation by collecting data for 24 consecutive weeks. However, natural limits imposed by our data collection techniques stymied our efforts. These natural limits included the proportion of people experiencing homelessness from outside of the city or province (lower than the overall population experiencing homelessness), COVID-19 outbreaks at the shelters, and the access (number of hours per week) we were provided at the shelters. Moreover, despite the main advantages of machine learning models—automatic pattern recognition and forecasting—the primary limitation of machine learning models is that they are not usable in testing hypotheses or proving relationships among variables. Therefore, the interpretation of the results for RQ1 and RQ3 can be further improved with the aid of a manual approach or clustering technique.

Several other possible enhancements could be carried out in this study. At this point, we have only identified the important factors predicting the *stays* or *leaves* and stay durations. Further study of the models should enable identification of the specific values of these factors in predicting a particular class label. In addition, advanced feature selection techniques can be used to enhance the identification of important factors. Another possible improvement is to perform experiments with the threshold used to decide whether a person stays or leaves shelters. Currently, we have used a static value of 8 weeks as the threshold after consulting domain experts. Moreover, special attention could be given to parameter tuning by using sophisticated methods such as GridSearchCV to enhance the performance of the models.

### Conclusions

The majority of the people experiencing homelessness in Thunder Bay were from outside of the city or province, according to the 2018 PiT count conducted by the city’s largest social service administrator. This high percentage had implications for service delivery, programming, and cost-effectiveness. The TBDSSAB sought to understand why so many of Thunder Bay’s population experiencing homelessness ultimately migrated from somewhere else. This study therefore presents the outcome of applying machine learning techniques to answer 3 RQs related to homelessness and migration in Thunder Bay. First (RQ1), we investigated the factors important for predicting whether someone chose to stay or leave shelters in Thunder Bay using a filter-based feature selection technique. The results for this RQ show that recent mental health support, availability of family or friends in Thunder Bay, and migrating for education are some of the most significant features in determining whether someone chose to stay or leave. Second (RQ2), we predicted the stay duration of individuals at shelters in Thunder Bay. For this RQ, we developed 8 machine learning models; the models based on ensemble learning techniques, such as RF and GBT, outperformed the other models with AUC values of 0.91 and 0.93, respectively. These results were obtained by performing filter-based and wrapper-based feature selection and using data augmentation techniques. Third and last (RQ3), we discovered the factors that were important for predicting the stay duration of migrants experiencing homelessness in Thunder Bay; these were home district, band membership, status card, previous district, and recent support for drug and alcohol use. In particular, we note the presence of 2 features related to Canada’s Indian Act: band membership and status card. Future research might focus more on these 2 features and ask why these 2 features are predictors of stay duration. Certainly, Indigenous peoples are overrepresented in the population experiencing homelessness in Canada, and this has been noted in Thunder Bay too [13,14].

## Appendix

Multimedia Appendix 1 Survey questions.

Multimedia Appendix 2 Details of the machine learning models and their hyperparameter settings.

## Abbreviations

AUC: area under the receiver operating characteristic curve
CV: cross-validation
GBT: gradient boosting tree
HIFIS: Homeless Individuals and Families Information System
KNN: k-nearest neighbor
MA: multiclass AdaBoost
MLP: multilayer perceptron
PiT: point-in-time
RF: random forest
RNN: recurrent neural network
RQ: research question
TBDSSAB: District of Thunder Bay Social Services Administration Board
TCPS 2: Tri-Council Policy Statement: Ethical Conduct for Research Involving Humans
TS: time stamp
XGBoost: Extreme Gradient Boosting

## Data Availability

The data sets generated and analyzed for this study are not publicly available because of the vulnerable nature of the participants, the small sample size, and the small population of the city in question. Data may be made available by the corresponding author upon reasonable request.
