# Supplementary material for: Understanding Why Many People Experiencing Homelessness Reported Migrating to a Small Canadian City: Machine Learning Approach With Augmented Data
Source: JMIR Form Res. 2023 May 2;7:e43511. doi: 10.2196/43511 (PMC10189624; doi:10.2196/43511)
Supplement: Multimedia Appendix 1 [file formative_v7i1e43511_app1.docx]

**Multimedia Appendix 1: Survey Questions**

- What is your home community?
- What is your previous community?
- What is your age?
- What is your sex?
- What is your ethnicity?
- What is the highest level of education you have completed?
- Do you have a Status Card?
- Do you have band membership?
- Was education/school a reason you came to Thunder Bay?
- Do you have family or friends in Thunder Bay?
- Was your family or were your friends a reason you came to Thunder Bay?
- Have you been incarcerated or to jail?
- Was court a reason you came to Thunder Bay?
- Did a fire, flood, or evacuation crisis force you to leave your home community?
- Was a medical appointment for yourself or a family member a reason you came to Thunder Bay?
- Have you been hospitalized in Thunder Bay?
- Was employment a reason you came to Thunder Bay?
- Did you find employment when you arrived in Thunder Bay?
- Are you currently employed?
- Was housing a reason you came to Thunder Bay?
- Was mental health support a reason you came to Thunder Bay?
- Have you received any mental health support recently?
- Was support for drug and/or alcohol use a reason you came to Thunder Bay?
- Have you received any support for drug and/or alcohol use recently?
- Are you barred from your community?
- Is cost a barrier to return to your community?
- Do you feel that you can't go back to your community for personal reasons?
- Do you feel you can't go back to your community for any other reasons?
