# Supplementary material for: Understanding Why Many People Experiencing Homelessness Reported Migrating to a Small Canadian City: Machine Learning Approach With Augmented Data
Source: JMIR Form Res. 2023 May 2;7:e43511. doi: 10.2196/43511 (PMC10189624; doi:10.2196/43511)
Supplement: Multimedia Appendix 2 [file formative_v7i1e43511_app2.docx]

**Multimedia Appendix 2: Details of the machine learning models and their hyperparameter settings.**

- The Decision Tree Classifier is capable of performing multi-class classification by building a tree with features as nodes [31]. The maximum depth of the decision tree was limited by setting it to 5. This was performed as a regularization technique to avoid overfitting. The quality of the split was measured based on the Gini impurity. When the split is pure, or in other words when a tree node contains samples belonging to the same class, the Gini score is zero.
- The SVM with a linear kernel was used as the next machine learning model to linearly separate the data points in the dataset into classes using a hyper-plane [32], [30]. Apart from the kernel, parameter ‘c’ represents the strength of the parameter regularization, and the class probability estimates were enabled by setting the probability to True.
- KNN is an algorithm that is used for both supervised and unsupervised learning methods. The mechanism of KNN in supervised learning is to predict the class label of a data point based on the labeled samples near that using a distance calculation [33]. In this work, the number of neighboring samples was declared as 6 and by default, equal weight was assigned to all the neighbors.
- Naive Bayes is a supervised learning algorithm built based on Bayes’ theorem and the “naïve” assumption of generating conditional dependence among each feature pair in the dataset. This model has shown higher performance in many real-world classification problems, even when training with a limited amount of data [34]. Here, we used the default parameters for training the model, such as priors kept as none to let the model adjust the prior probabilities of the class according to the dataset.
- Random Forest is an ensemble learning method that uses a collection of decision trees. The performance of each decision tree which is built from different sub-samples of the dataset is averaged in producing better results for the random forest [35]. This model has proved its ability to minimize overfitting compared to decision trees. The size of subsamples is decided by the number of trees we specify as a parameter. Here, the number of trees was set to 500 and by making bootsratp=True, we disabled the use of the whole dataset when building each tree. Furthermore, we have added randomness to the model to allow random feature selection when building the trees.
- Logistic Regression is a linear model which is used for classification. This model generates probabilities for each outcome to indicate their corresponding class using the logistic function [36]. The newton-cg was used as the solver to support optimization in a multi-class problem. The model regularization was enabled by default and the newton-cg supports the l2 regularization.
- MLP is utilized as a non-linear classification model that requires numerous hyper-parameter tunings [37]. The selection of values for hyper-parameters severely affects the results of the model. However, we performed a random hyper-parameter search which is recognized as comparatively effective due to the higher computational burden of a grid search [22] and finalized the model with the psarameters given in Table I.
- Gradient Boosting is also an ensemble learning method where several base models are combined to produce generalizability in both classification and regression problems [35]. We used 500 as the number of boosting stages to avoid overfitting and enabled randomness when building the tree.

References

22. B. VanBerlo, M. A. S. Ross, J. Rivard, and R. Booker, “Interpretable machine learning approaches to prediction of chronic homelessness,” *Eng. Appl. Artif. Intell.*, vol. 102, no. April, p. 104243, 2021.

30. A. Vabalas, E. Gowen, E. Poliakoff, and A. J. Casson, “Machine learning algorithm validation with a limited sample size,” *PLoS One*, vol. 14, no. 11, pp. 1–20, 2019.

31. “scikit Learn - Decision Trees,” *License), scikit-learn developers (BSD*, 2022. [Online]. Available: https://scikit-learn.org/stable/modules/tree.html#tree. [Accessed: 25-Apr-2022].

32. “scikit-learn Support Vector Machines,” *scikit-learn developers (BSD License)*, 2022. [Online]. Available: https://scikit-learn.org/stable/modules/svm.html#svm-classification. [Accessed: 29-Apr-2022].

33. “scikit-learn Nearest Neighbors,” *scikit-learn developers (BSD License)*, 2022. [Online]. Available: https://scikit-learn.org/stable/modules/neighbors.html#classification. [Accessed: 29-Apr-2022].

34. “scikit-learn Naive Bayes,” *scikit-learn developers (BSD License)*, 2022. [Online]. Available: https://scikit-learn.org/stable/modules/naive_bayes.html#gaussian-naive-bayes. [Accessed: 29-Apr-2022].

35. “scikit-learn Ensemble Learning,” *scikit-learn developers (BSD License)*, 2022. [Online]. Available: https://scikit-learn.org/stable/modules/ensemble.html. [Accessed: 01-May-2022].

36. “scikit-learn Logistic Regression,” *scikit-learn developers (BSD License)*, 2022. [Online]. Available: https://scikit-learn.org/stable/modules/generated/sklearn.linear_model.LogisticRegression.html. [Accessed: 01-May-2022].

37. “scikit-learn Neural Networks,” *scikit-learn developers (BSD License)*, 2022. [Online]. Available: https://scikit-learn.org/stable/modules/neural_networks_supervised.html. [Accessed: 01-May-2022].
